# Improving mental health care in depression: A call for action

**DOI:** 10.1192/j.eurpsy.2023.2434

**Published:** 2023-08-03

**Authors:** Julia Eder, Geert Dom, Philip Gorwood, Hikka Kärkkäinen, Andre Decraene, Ulrike Kumpf, Julian Beezhold, Jerzy Samochowiec, Tamas Kurimay, Wolfgang Gaebel, Livia De Picker, Peter Falkai

**Affiliations:** 1 Department of Psychiatry and Psychotherapy, LMU University Hospital, LMU Munich, Munich, Germany; 2 Graduate Program “POKAL - Predictors and Outcomes in Primary Care Depression Care” (DFG-GrK 2621), Munich, Germany; 3 Collaborative Antwerp Psychiatric Research Institute (CAPRI), University of Antwerp, Antwerp, Belgium; 4 Université Paris Cité, GHU Paris (Sainte Anne hospital, CMME) & INSERM UMR1266, Paris, France; 5 Global Alliance of Mental Illness Advocacy Networks-Europe, Brussels, Belgium.; 6 EUFAMI, the European Organisation representing Families of persons affected by severe Mental Ill Health, Leuven, Belgium; 7 Norfolk and Suffolk NHS Foundation Trust, Norwich, UK, University of East Anglia, Norwich, UK; 8 Department of Psychiatry, Pomeranian Medical University, Szczecin, Poland; 9 North-Central Buda Center, New Saint John Hospital and Outpatient Clinic, Buda Family Centered Mental Health Centre, Department of Psychiatry and Psychiatric Rehabilitation, Teaching Department of Semmelweis University, Budapest, Hungary; 10 Department of Psychiatry and Psychotherapy, Medical Faculty, Heinrich-Heine-University, Düsseldorf, WHO Collaborating Centre DEU-131, Germany; 11 Max-Planck Institute of Psychiatry, Munich, Germany

**Keywords:** policy paper, Future trends inpsychiatry, political framework, depressive disorders

## Abstract

Depressive disorders have one of the highest disability-adjusted life years (DALYs) of all medical conditions, which led the European Psychiatric Association to propose a policy paper, pinpointing their unmet health care and research needs. The first part focuses on what can be currently done to improve the care of patients with depression, and then discuss future trends for research and healthcare. Through the narration of clinical cases, the different points are illustrated. The necessary political framework is formulated, to implement such changes to fundamentally improve psychiatric care. The group of European Psychiatrist Association (EPA) experts insist on the need for (1) increased awareness of mental illness in primary care settings, (2) the development of novel (biological) markers, (3) the rapid implementation of machine learning (supporting diagnostics, prognostics, and therapeutics), (4) the generalized use of electronic devices and apps into everyday treatment, (5) the development of the new generation of treatment options, such as plasticity-promoting agents, and (6) the importance of comprehensive recovery approach. At a political level, the group also proposed four priorities, the need to (1) increase the use of open science, (2) implement reasonable data protection laws, (3) establish ethical electronic health records, and (4) enable better healthcare research and saving resources.

## Treatment of depression today

### The reality of diagnosing and treating depression

In Europe, almost one citizen in five is diagnosed with depression. The 12-month prevalence of depression varies between different regions but is approximately 6% across all countries. The first depressive episode typically occurs in a period between adolescence and the mid-40s [1]. The European Study of the Epidemiology of Mental Disorders showed that only 36.8% of participants with an affective disorder and 20.6% with an anxiety disorder reached out to health services for help. Of the participants who sought help, 20.7% received no therapy to alleviate their mental health problems [2]. Depressive disorders have a peak prevalence between the ages of 25 to 35. They are referred to as complex disorders because their etiology involves an interaction of genetic and environmental risk factors. Despite the availability of evidence-based treatments, only 45% of patients with depression recover permanently [3]; According to the World Health Organization, unipolar depression and other mental disorders on average lead to a significant functional impairment more debilitating than chronic diseases like diabetes [2]. Compared with the general population, people with mental illnesses such as depression are in poorer health and their life expectancy is lower [3], showing a need for improvement of diagnostic capabilities, as well as expansion of care for the affected.

The associated reasons are outlined in the first part of this policy paper below and are used as the basis for European Psychiatric Association (EPA) action points which aim to ensure that practitioners will soon be better able to help affected individuals better (Table 1). The second part of the paper presents developments that may revolutionize the treatment of depression in the coming years but are not yet established in clinical care.

**Table 1.** Improving clinical care now. The table shows the recommendations by the task force that can be implemented to improve clinical care now

The EPA asks that the following steps be taken now to improve clinical care:
1. Every patient seen in a non-specialist setting, e.g. by a GP, needs to be evaluated with brief screening instruments to detect anxiety disorders, depression, and substance abuse disorders.
2. Every patient with a diagnosis of any mental disorder should undergo a physical evaluation, including a neurological examination (including an EEG), blood tests (including blood glucose, a complete blood cell count, and thyroid hormones), electrocardiogram and brain imaging (computed tomogram or MRI), if possible. This evaluation is especially important if the patient has physical symptoms. Pathological findings need to be followed up and treated.
3. Once a probable diagnosis of depression is made, a patient needs to be seen at least once by a psychiatrist to confirm the diagnosis. The patient should be referred to other medical disciplines as needed.
4. Once the diagnosis and severity of the symptomatology have been evaluated, CBT needs to be started and the patient needs to receive 12 to 15 therapeutic units within 3 to 6 months. If the patient does not achieve full recovery by the end of this period, psychotherapy should be continued, and other methods should be offered.
5. Pharmacotherapy or possible additional somatotherapy needs to be started as soon as possible. In the case of only partial recovery or other risk factors, pharmacotherapy needs to be continued and monitored in the long term because otherwise, the patient will be at high risk of developing a relapsing course.
6. All of the above steps must follow the evidence-based principles laid down in the respective guidelines.

This paper focuses on depressive disorders to pinpoint their unmet health care and research needs.

### Diagnosing depression and its comorbidities

To date, health systems and research facilities have failed to adequately meet the needs of patients requiring treatment for depression [4]. Mental illnesses affect all age groups and social classes, albeit with varying frequency and severity. Moreover, in general practice physical illness that occurs either independently of or in the context of treatment of mental illness is often also inadequately treated [5]. For example, although cardiovascular diseases have a higher prevalence in patients with psychiatric disorders as well as depression, this group of patients has a below-average detection [6].

Many subjects with major depressive disorder initially present to their general practitioner (GP) [7]. One study found that GPs correctly diagnosed depression in only 47.3% (95% CI, 41.7 to 53.0%) of cases [8]. Other psychiatric diseases are also currently underdiagnosed in primary care [9]. This underdiagnosis leads to increased morbidity and mortality, worse functional outcome, higher suicide rates, and more frequent use of unnecessary diagnostic procedures [10]. After being diagnosed with depression, a patient should see a psychiatrist, who will determine a therapeutic regimen and work in close cooperation with other medical disciplines. However, if a psychiatrist is not available, seeking assistance from other qualified mental health practitioners can be a suitable alternative.

The importance of the above issues was described in a Lancet commission paper on the global challenges regarding depression [11] so the present paper will focus on the European perspective.

### Treating depression with evidence-based approaches as early as possible

If depression is not treated, only about 8 to 18% of people achieve remission [12]. Furthermore, stigmatization of mental disorders aggravates psychopathology and impedes symptom improvement across all mental disorders [13, 14]. Thus, selecting the correct treatment for depressive disorders is an important task for psychiatrists.

#### Psychotherapy: when, which one, and for how long?

Choosing the most appropriate treatment for depression depends on the severity of the symptoms. For mild symptoms, psychotherapy alone can be sufficient with 2–5 sessions of cognitive behavioral therapy (CBT). However, if the symptoms are moderate, it is recommended to combine 12–15 sessions of CBT with pharmacotherapy, such as antidepressants, according to the National Institute for Health and Care Excellence [15]. For severe episodes of depression, it is recommended to offer a combination of 12 to 15 CBT sessions and appropriate pharmacotherapy [16].

Although psychotherapy can be an effective treatment, the long duration of therapy and a limited number of psychotherapists may cause patients to wait for 2 to 5 months before their first session [17], whereas long waiting periods are associated with more unfavorable outcomes [18]. Other psychotherapy methods such as psychodynamic therapy or integrative methods can also be effective in treating mental health disorders. To bridge this gap and increase the availability of psychotherapy, E-mental health methods need to be incorporated into treatment [19]. These methods encompass a wide range of digital technologies and platforms designed to deliver mental health interventions remotely. Examples include Internet-based cognitive-behavioral therapy (iCBT), mobile applications for self-management, virtual reality therapy, and online support groups. E-mental health methods can enhance the availability and accessibility of psychotherapy, reaching individuals who may face barriers to traditional in-person therapy, such as geographical limitations, stigma, or lack of resources. It is important to recognize that e-mental health methods are not meant to replace face-to-face therapy but rather to complement and expand the options available to individuals seeking mental health support [19, 20].

It is also important to note that the interpersonal relationship between the patient and therapist can greatly impact the success of therapy. Building a positive therapeutic relationship based on trust and mutual respect can lead to better outcomes and a more positive therapy experience for the patient. Ultimately, the best treatment plan should be based on the individual’s unique circumstances, preferences, and the quality of the therapeutic relationship [21].

#### Pharmacotherapy/Somatotherapy: when, which one, and for how long

Antidepressants have been shown to be effective in treating moderate to severe depression [15]. Medication can be an effective treatment for mental health conditions, providing relief to patients suffering from symptoms. As with any medical treatment, adverse effects can occur with drug treatment that may cause patients to discontinue their medication. To avoid this, physicians must educate patients regarding possible side effects, monitor them, and switch them to another medication, increase the dosage, or start augmentation if there is only minimal improvement even in the absence of side effects after 2–3 weeks of treatment [22]. To minimize relapse and treatment failure, maintenance therapy and monitoring for a minimum of 6 months after remission are recommended, followed by a gradual tapering of the antidepressant. Prolonged treatment can lead to overtreatment, while premature discontinuation may cause relapses. However, the determination of the appropriate treatment duration is an area that requires further research [23].

However, it often takes several attempts to find the most suitable drug because current treatment regimens do not specifically fit the needs of individual psychiatric patients; this situation can lead to frustration and underscores the need for personalized psychiatry [24]. It is particularly challenging to find a suitable regimen for treatment-resistant depression [25]. Pharmacotherapy can be complemented or even replaced by brain stimulation methods such as electroconvulsive therapy (ECT) or repetitive transcranial magnetic stimulation (rTMS) [26, 27]. Incorporating these treatments may involve challenges such as stigma, and anesthesia concerns for ECT. Like ECT, rTMS is typically provided in specialized clinics for patients with chronic depression, and accessing these specialized treatments may require appropriate referrals. Careful consideration of individual patient needs is essential when considering these alternative options.

## Treatment of depression tomorrow

### The future – methodology

We initiated a task force consisting of nine EPA board members. Through a series of five meetings, the task force engaged in in-depth discussions, carefully considering various aspects of the topic. The discussions were documented and analyzed thematically to capture the key insights and perspectives shared by the task force members [28]. During five meetings, the group exchanged ideas about the future of psychiatry until they felt that they had discussed all relevant topics in sufficient detail. This approach aimed to provide a comprehensive understanding of the future of psychiatry based on expert perspectives and collaborative deliberation.

All participants in the study gave oral consent to participate and to the publication of the results. Because of the participants’ professional status, ethics committee approval was not deemed necessary.

### Diagnosing and treating depression tomorrow

Currently, mental health care faces limitations in integrating advanced technology due to fragmented care systems and limited resources. Socioeconomic, stigma and cultural disparities further restrict access to care. To overcome these challenges a multi-faceted approach involving policy changes, increased funding, education, and collaboration between various stakeholders as well as continued research is necessary.

**Figure 1.**
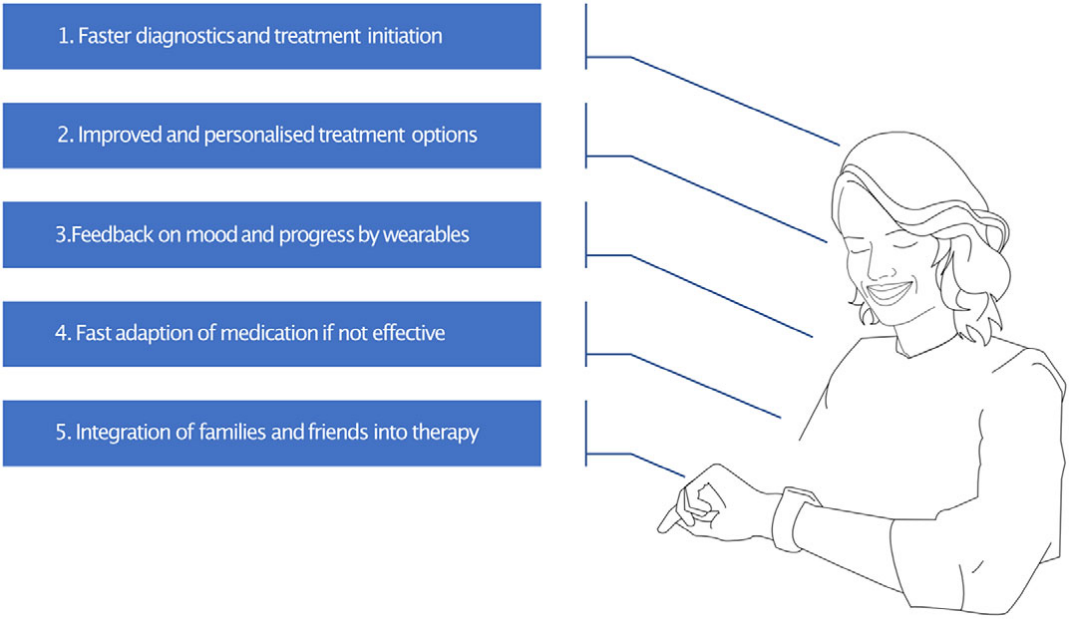
The figure shows an individual with unipolar depression in 10 years and positively highlights the aspects that have changed for the better.

Looking towards the future, our vision for mental health care is transformative. We envision a seamless and personalized experience that empowers individuals to take control of their mental well-being (Figure 1). When someone seeks help for depression or other mental health concerns (e.g., from their general practitioner), they will reach a comprehensive and integrated system of care before symptoms become debilitating.

Advanced algorithms and artificial intelligence will be used to analyze a wide range of patient data, including medical history, lifestyle factors, and environmental influences, to develop a personalized treatment plan. Wearable technology and other connected devices will allow patients to monitor their own progress and receive real-time feedback on their mental health status. Imaging, HRV, and EEG data can be added if the algorithm needs more information to make a prediction about an individual patient. Additional blood can be drawn for metabolomics, proteomics, transcriptomics, and genomics analyses, the results of which can be entered into the system to predict which treatment will be the most effective and have the fewest adverse effects.

Psychotherapy and other evidence-based treatments will be tailored to each patient’s unique needs and preferences. Patients will have access to a variety of digital resources, including apps and online communities, that can help them stay motivated and engaged in their treatment. Lifestyle and nutrition advice is also provided, and follow-up is implemented with wearables. The patient can obtain information about reducing their personal risk of recurrence of depression in the peripartum period if they want to have children. The stigma around mental illness will be greatly reduced, as society becomes more accepting of mental health issues. Schools and workplaces will prioritize mental well-being, with policies and programs that support their employees and prevent burnout.

Also, in the future mental health care providers will stay up-to-date with the latest research and developments in the field, including advances in technology and evidence-based practices. Finally, providers will continue to enhance their collaborative efforts with other healthcare professionals and support systems, such as family members or friends, to ensure even greater levels of comprehensive and coordinated care in the future.

## Elements enabling a radical change in psychiatry

### Increased awareness of mental illness in primary care settings

The high rate of psychiatric comorbidity in primary care patients stresses the need to provide psychiatric care in primary and specialty care settings and to increase cooperation between GPs and psychiatrists [29]. Algorithms are needed to assist GPs in recognizing depression and initiating treatment in patients who are likely to have a favorable outcome. A consultation with a psychiatrist should be suggested in cases that are likely to be complex, i.e., patients with poor clinical outcomes after treatment of their medical problems and psychiatric disease in primary care and those with a poor prognosis for social and occupational functioning.

Early psychiatric assessment and treatment recommendations by liaison psychiatrists help clarify cases and facilitate access to appropriate interdisciplinary care [30]. Psychiatric counseling should quickly lead to low-threshold support and adequate treatment. Providing early training and psycho-education to family members can enhance support and lower the risk of escalation [31].

### The development of novel (biological) markers

Precision psychiatry can deliver a tailored diagnostic concept and individualized treatment for heterogeneous clinical conditions such as depression by combining genetic, behavioral, cognitive, and biological markers [32]. Several trophic factors were found to be altered in patients with depression compared with controls [33]. The most studied cytokines in psychoneuroimmunology are interleukin (IL)-6, tumor necrosis factor (TNF) alpha and beta, IL-1b, and interferons. The dysregulation of interleukins [34], neurotrophic factors, and hormones among other molecules, has been associated with schizophrenia and other mental diseases. However, despite extensive research, definitive etiological pathways linking the molecules to these disorders have yet to be discovered [35].

Various genetic loci have been identified that increase the risk of becoming mentally ill. Nevertheless, this abundance of knowledge has not been translated into routine psychiatric care. Integrating findings from research knowledge into clinical care remains an important challenge for the future, in particular, because current normal routine markers do not allow reliable diagnosis of psychiatric diseases [36, 37].

### Machine learning supported diagnostics, prognostics, and therapeutics

The increasing use of technology in psychiatric assessments and treatment will lead to decentralization and broader access to therapeutic services. These developments will enable us to progressively destigmatize psychiatric illnesses and will improve psychiatric care in rural areas and developing and emerging countries [38]. Predictive psychiatry, which often relies on machine-learning algorithms, will play an important role as a pillar in the prevention of relapse and re-emergence of an existing psychiatric illness [39, 40]. While machine-learning algorithms are often employed in predictive psychiatry studies to forecast treatment outcomes, their clinical utility is still lacking. The practical application of this method is hindered by its prohibitive cost, particularly due to the requirement of MRI scans for models with good accuracy scores. As a result, machine learning has not yet achieved the desired level of effectiveness in the field of precision psychiatry [39].

### Implementing therapeutic apps for electronic devices into everyday treatment

Medical care through smartphone apps and associated sensors is at the cusp of moving from being a topic of research to being implemented in outpatient care. Some apps already exist, that help patients track their symptoms, review and apply content from psychotherapy and perform cognitive training and also support patients in adhering to their medication [41, 42].

Health apps can improve the diagnosis and prevention of diseases, provide patients with psychoeducation in the face of a shortage of therapists and monitor progress during therapy [41, 43]. Patients may share very sensitive data via apps, which may also be connected to other sensors such as a smartwatch, so strict data protection and data security rules are needed to reduce the risk of data violations [44].

### Plasticity-promoting somatotherapy – a new generation of treatment options

Classical psychedelics typically include lysergic acid diethylamide, psilocybin, and *N*,*N*-Dimethyltryptamine [45]. These substances interact with 5-HT2A receptors, which are predominantly located in the caudate, nucleus accumbens, olfactory tubercule, hippocampus, and the pyramidal cells of the neocortex. The anesthetic ketamine is also a hallucinogen in the broader sense, although it affects the glutamatergic system via the N-methyl-D-aspartate receptor [46]. Hallucinogens are thought to disrupt engrained thought patterns by counteracting acquired synaptic deficits, neural atrophy, and loss of connectivity in the frontal neocortex through enhanced neuroplasticity, thus allowing ruminations and negative thought spirals to be disrupted [47]. However, there is conflicting evidence about the persistence of those effects, especially in the case of ketamine [48].

Also, brain stimulation techniques like rTMS and ECT are able to induce plasticity and modulate dysfunctional brain network activity [49]. New accelerated protocols are being developed and implemented in rTMS treatment regimens along with individualized procedures like fMRI-informed neuronavigated or EEG-feedback closed-loop stimulation, to improve response rates [50].

### Comprehensive recovery approach

In addition to the important interventions and supportive technologies described above, treatment should seamlessly incorporate all relevant life areas of the patient with the aim to not only achieve recovery from the current episode but also build up resilience to support better future overall health. Particularly in depression, patients have a higher risk of relapse if they have only a limited social network [51, 52]. Holistic treatment requires stigma-free contact with the patient’s employer to enable changes in the workplace; training in lifestyle skills (e.g., nutrition and exercise); close involvement of the patient’s social contacts (e.g., partner, family); and consideration of the patient’s cultural background (cultures differ across Europe and patients might be additionally shaped by migration) [53].

Patients with depression and their relatives and social networks should be increasingly integrated into treatment and research. Further, therapy requires coordination between the treating doctors and the therapists involved.

Studies have provided evidence that besides social participation, diet, and physical activity are beneficial for mental health and are cost-effective [54]. Furthermore, psychiatrists, GPSs, and therapists should regularly receive super- and intervision to steadily improve the quality of care [55].

## Political framework

### Open Science to accelerate progress

Open Science encompasses various aspects of the scientific working process. Its central idea is that everyone should be able to contribute to research [56]. Consequently, scientific publications should be publicly accessible without charge, and data sets used should be published to ensure better replicability and reproducibility in general [57]. Machine learning and genetic data analysis usually require very large data sets. To benefit from synergies, Open Science is an important element because it allows the necessary size of data sets to be achieved more easily and can also provide external validation data sets [58].

However, Open Science has also been criticized because it increases the number of retractions and articles containing errors, which pollute the quality of journals [59]. Open-source projects are often run on a voluntary basis so correcting faulty processes takes longer [60].

### Reasonable data protection laws

As the world becomes increasingly technological, data streams can be analyzed much more easily. However, when personal data are combined with large external data sets, data scientists can derive information about individuals that the person may want to keep confidential or may not even be aware of [61]. The provision of new technologies for gigantic data sets and analytical methods for evaluating them has given rise to the vision of deep phenotyping. Phenotyping is a necessary building block for tracing psychiatric diseases back to their biological correlates. However, the further one explores this issue, the more ethical questions arise that need to be addressed [62]. Therefore, to maximize benefits while minimizing harm to study participants, in 2018 the European Union introduced the General Data Protection Regulation (GDPR). The GDPR also aims to harmonize regulations across Europe [63]. Although it does not solve all data protection-related issues, the GDPR does provide a framework for harmonizing data protection laws and simplifying the implementation of multicenter studies across the European Union. On the one hand, data regulation should protect participants’ data, but on the other, it should also provide feasible strategies to facilitate data acquisition and processing to allow meaningful scientific research to be performed [63]. We recommend establishing an interdisciplinary task force to specifically work on solving these issues.

### Ethical electronic health records

An electronic health record (EHR) is a digital database that not only contains a patient’s personal data but also archives longitudinal data about diseases, diagnostic procedures, treatments, and responses to therapy. Consequently, the most obvious advantage of EHRs is that they provide easy accessibility to medical data for medical professionals in case a patient moves and thus changes doctors [64]. The introduction of EHRs can improve the safety, quality, and efficiency of medical systems over the long term because the improved flow of information means that redundant examinations can be avoided [65]. A public funding source is needed to create an ethical EHR, ensure consistent standards, develop less error-prone interfaces, and avoid conflicts of interest so that the focus is always on patient well-being and evidence-based medicine [66].

To develop an EHR that can meet these requirements, computer scientists and doctors who are familiar with the development and requirements of such an application must work together to prevent regulations, especially those that may be rather detrimental to patient welfare and beneficial to other parties [67]. With the constant progress of scientific knowledge, the contents of an EHR must also be regularly re-evaluated and improved. Consequently, independent specialists must periodically accredit EHRs, including testing them for malfunctions and checking whether decision support systems are still working according to current medical standards and whether the EHRs meet current safety standards. Regular peer review of the medical content and source codes could be used to raise awareness of content that is outdated or malfunctioning [68].

In addition to establishing a uniform system that avoids the potential for creating multiple error-prone interfaces, clear and mandatory security standards are also needed. The lack of such an infrastructure is also currently hampering the establishment of a Europe-wide EHR [69].

### Enabling better healthcare research and saving resources

Open Science, reasonable data protection, and a standard EHR will enable better healthcare research by providing large datasets and thus facilitating the evaluation of treatment strategies and the early diagnosis and prevention of diseases. Specifically tailored therapeutic approaches can be developed to offer patients the best treatment, which ultimately translates into the effective implementation of precision medicine [70]. Currently, only 9% of patients with unipolar depression being treated in primary care receive adequate treatment. If patients with unipolar depression were diagnosed more reliably and treated more effectively, healthcare costs would decrease. The ratio of means costs is 2.28-fold higher in adults with depression than in non-depressed adults [71]. In addition, studies evaluating excess costs largely do not assess indirect costs, such as those caused by reduced productivity [71].

The US Preventive Services Task Force currently recommends broad screening in all adults, regardless of risk factors, because early treatment improves patient symptomatology [72].

Furthermore, it is important to acknowledge the critical role of patient engagement in depression treatment. Despite having access to various treatment options, a significant barrier for individuals with depression is the motivation to actively participate in their treatment. Addressing this issue requires a multifaceted approach that considers strategies to improve patient engagement. Psychoeducation, shared decision-making, and tailored interventions are essential for optimizing treatment outcomes [73].

The potential for reduced healthcare costs with improved diagnosis and treatment of unipolar depression is a valid consideration, further research is needed to comprehensively evaluate the cost-effectiveness of these interventions.

## Limitations

This task force has uncovered revolutionary ideas for the future of psychiatry. Nevertheless, it has some limitations. For example, it may have had a selection bias because only a small subgroup of EPA members participated. The specialists also highlighted the importance of their personal interest in the presented topic and their need to feed their own enthusiasm and interest in learning. This aspect questions the external validity of the study. Nevertheless, we assume that the group covered most of the relevant topics in their group discussions.

## Summary: the EPA is moving the needle

**Figure 2.**
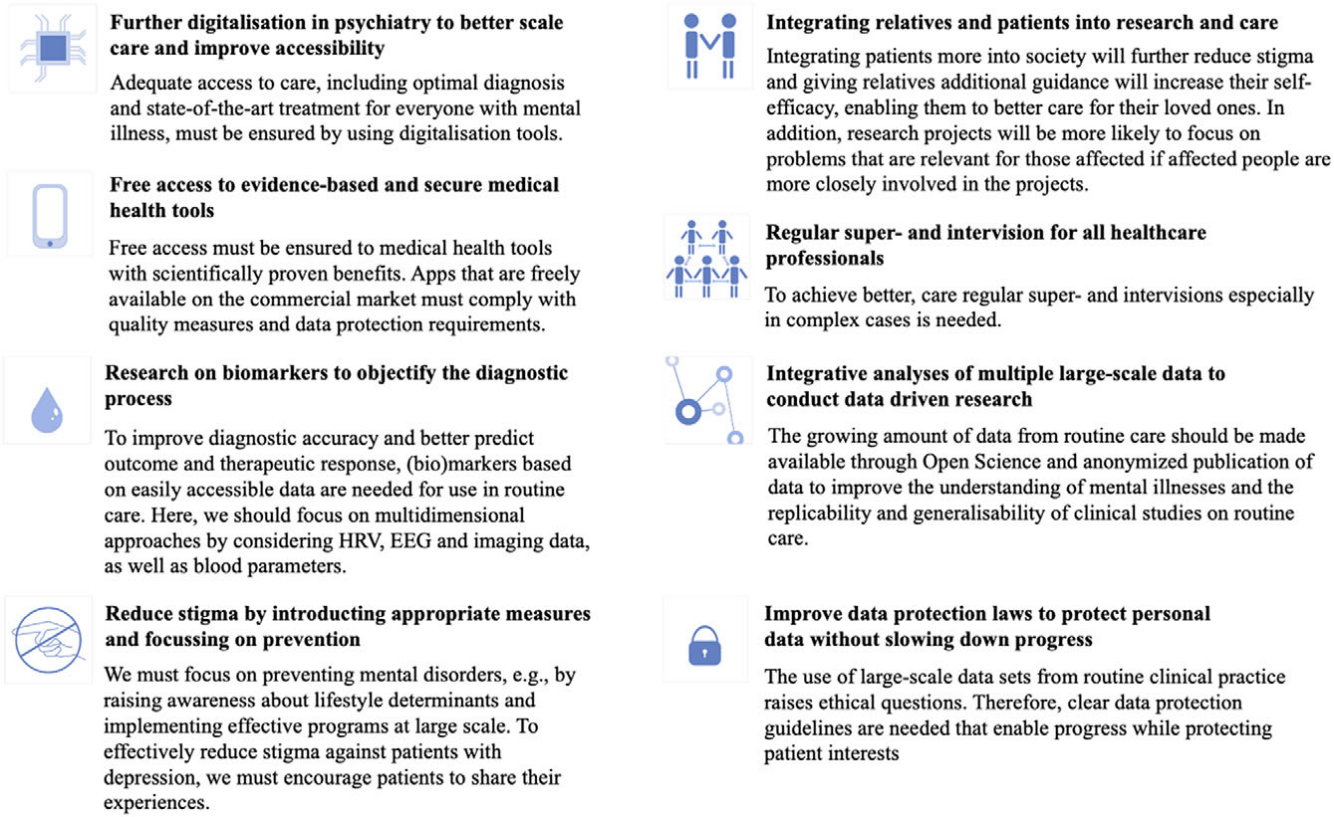
Joint action on depression in Europe. The diagram shows the recommendations developed by the task force comprising members of the European Psychiatric Association.

To significantly improve the quality of care for people with mental illness by immediately increasing recovery and reducing mortality, the EPA proposes the following action points (see Figure 2): 1. Digitalise psychiatry. 2. Ensure access to medical health apps. 3. Develop suitable (bio-) markers to facilitate the diagnostic process. 4. Implement preventive pipelines, reduce stigma, and raise awareness. 5. Integrate patients and their relatives and social networks into care and provide access to knowledge and training to enhance their coping and care skills. 6. Provide regular super- and intervision for all healthcare professionals. 7. Perform integrative analyses of large-scale data from multiple sources to ensure replicability and generalisability of research findings on routine care. 8. Create clear and usable data protection guidelines.
